# Comparison of Anatomical Maxillary Sinus Implant and Polydioxanone Sheets in Treatment of Orbital Floor Blowout Fractures: A Retrospective Cohort Study

**DOI:** 10.3390/jfb16060204

**Published:** 2025-06-02

**Authors:** Benjamin Walch, Alexander Gaggl, Gian Battista Bottini, Johannes Hachleitner, Florian Huber, Hannes Römhild, Martin Geroldinger, Maximilian Götzinger

**Affiliations:** 1 Department of Oral and Maxillofacial Surgery and Center for Reconstructive Surgery, University Hospital of the Private Medical University Paracelsus, 5020 Salzburg, Austria; b.walch@salk.at (B.W.); a.gaggl@salk.at (A.G.); g.bottini@salk.at (G.B.B.); j.hachleitner@k.at (J.H.); fl.huber@salk.at (F.H.); h.roemhild@salk.at (H.R.); 2 Team Biostatistics and Big Medical Data, IDA Lab Salzburg, Paracelsus Medical University, 5020 Salzburg, Austria; martin.geroldinger@pmu.ac.at; 3 Research Program Biomedical Data Science, Paracelsus Medical University, 5020 Salzburg, Austria

**Keywords:** orbital fracture, orbital implant, trauma surgery, biocompatible material, ocular surgery, ophthalmologic diagnostic techniques, blowout fractures, double vision, enophthalmos, maxillary nerve, maxillary sinus

## Abstract

Background: Orbital floor blowout fractures (OFBF) can have serious consequences for the patient. Selecting the right treatment method and materials is essential. Krenkel’s maxillary sinus implant has been used successfully for more than 40 years in clinical practice. The aim of this study was to evaluate the long-term outcome of this implant compared to polydioxanone (PDS) sheets. Material and methods: This retrospective study examined a cohort of 82 OFBF patients over a seven-year period. Clinical and geometric data were collected. Defect size, location, and the volume of the herniated tissue were measured from conventional computer tomography (CT) or cone beam computer tomography (CBCT) scans. The relationship between ophthalmologic rehabilitation and treatment modality was analyzed using logistic regression. Results: The study included 82 patients, 28% female and 72% male, with a median age of 45.2 years. Defect size and hernia volume correlated with preoperative ophthalmological symptoms. At follow-up, 14.8% in the implant group and 28.6% in the PDS group showed mild visual impairment, with no severe diplopia. Conclusions: Our results suggest this method is a reliable and effective solution for repairing OFBFs and ophthalmologic rehabilitation. However, further research in a clinical controlled trial is needed.

## 1. Introduction

The bony orbit is an anatomically complex three-dimensional region that contains the bulbus oculi and protects it from mechanical forces. Seven bones form this pyramid-shaped cavity. The walls vary in their thickness and resistance [1].

The orbital floor and the medial orbital wall can be very delicate. They often break in midface trauma [2]. Facial trauma can have serious consequences for this delicate and functional crucial region, like enophthalmos, impaired eye movement, and diplopia being key symptoms. The latter two can be caused by entrapment of herniated tissue [3].

If not treated in time, damage to the external eye muscles, especially the rectus inferior muscle, followed by scarring of those structures, can lead to permanent eye movement disorders, and double vision. Depending on the severity, this can lead to morbidity and disability for the patient. Double vision, within 20-degree eye movement, results in a severe impairment in daily life activities, including driving and operating machines. Thus, timely surgical release of entrapped external eye muscles is imperative in treating orbital fractures [4,5]. In addition to a possible dysfunction of extraocular muscles, the cause of double vision can also be a fracture-related asymmetry in the position of the globe, which results in a misalignment of the visual axis between the two orbits. The paper-thin and fracture-prone posteromedial transition zone, from the floor to the medial wall of the orbit, is crucial for the anteroposterior position of the bulbus oculi. If fractured, this key region requires an anatomically precise reconstruction to correct the position of the globe [5].

A fracture of any part of the orbital walls without involving the orbital rim is called a pure orbital blowout fracture [6]. Orbital floor blowout fractures (OFBF) may be managed either conservatively or surgically. It is widely accepted that specific clinical findings necessitate surgical treatment. These include visible enophthalmos, greater defect sizes, severe fragment dislocation, restricted ocular motility associated with diplopia and prolapse, and incarceration of muscular or large amounts of orbital soft tissue.

OFBFs are commonly reconstructed using alloplastic implants to cover the orbital defect; however, the underlying native bone is often not incorporated into the repair [7]. Many resorbable alloplastic patches are available for small to medium-sized OFBFs, while large and complex fractures are typically reconstructed with titanium meshes [8,9,10].

Evidence indicates that resorbable materials used as patches, sheets, membranes, and plates are highly effective in clinical applications. Some even demonstrated favorable results in treating larger OFBFs with defect sizes exceeding 2 cm^2^ or 2.5 cm^2^, representing thresholds commonly considered the upper limit for those materials. On average, defects of that size equal approximately one-third to half of the total area of the entire orbital floor [8,11,12]. In a national French survey published in 2023, the materials used for orbital floor fracture repair were examined, and the PDS sheet was found to be the most used resorbable reconstruction material, especially for small defects [13]. Evidence shows PDS sheets are among the most common treatment options and provide a good therapeutic method for small- and medium-sized defects related to postoperative enophthalmos and diplopia, while porous polyethylene and titanium mesh are preferred for larger defects [13,14].

PDS sheets are available in thicknesses of 0.15 mm, 0.25 mm, and 0.5 mm. After just three weeks, the PDS has lost half of its strength and is dissolved after six months. Complete biodegradation typically occurs within six months. During this period, tissue healing leads to the development of a fibrous scar, which assumes a stabilizing role as the PDS material resorbs [15].

Reconstruction of the orbital floor appears to be more precise when using a 0.25 mm PDS film compared to a 0.5 mm film, potentially due to the lower flexural stiffness of the thinner material [15].

Alternatively, the inferiorly displaced orbital bone can be repositioned and stabilized using an osteosynthesis plate for orbital floor reconstruction. Additionally, autologous bone grafts from other donor sites can be viable alternatives for orbital floor reconstruction. For medium-sized up to large and complex OFF, reconstruction can be achieved by repositioning the displaced orbital bone through a transantral approach. This method involves filling the maxillary sinus to create an anatomically appropriate support for the orbital floor [7,16,17].

Studies on orbital fractures utilizing the balloon technique for reconstruction are limited in the literature. Frequently, medical devices intended for other purposes, such as Foley catheter balloons, are repurposed for this off-label use [18,19]. Despite the potential for complications, including rupture or malposition of these devices, they have proven effective in restoring the anatomical volume and contour of the orbit following fractures of the orbital floor and medial wall [18,19,20,21,22,23,24].

**Figure 1:**
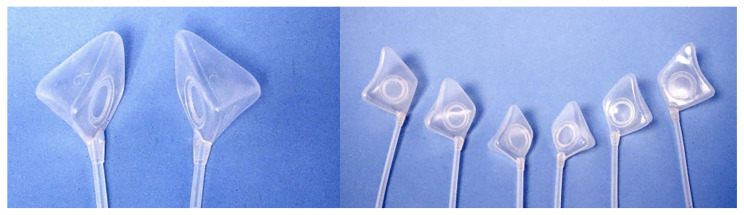
The three different-sized anatomical sinus endotheses are for the right and left maxillary sinus on the left view from the top and the right view from the medial.

In 1984, Krenkel designed an anatomical maxillary sinus implant by measuring cadaveric specimens. His data showed that the maxillary sinus has a relatively uniform shape, with the only major difference among the specimens being the volume [25]. Thus, he designed three different-sized casts for large, medium, and small sinuses (Figure 1).

More than 40 years later, those casts are still used for creating in-house crafted sinus balloons. However, large-scale comparative research on those devices has never been conducted. Published preliminary results, case series, and the trust of the clinicians in Salzburg using those devices based on over 40 years of long-term experience and perceived good results, are the basis for applying the implant in treating OFBF. It remains the go-to tool for treating medium to large-sized OFBF without indication for the repair of the medial wall at our facility [7,25].

The sinus implant features a thin, pliable, anatomically accurate silicone body and a filling tube to inflate or deflate the balloon through a small osteotomy window created in the lower nasal passage connecting the nasal cavity and the maxillary sinus. The nasal approach enables the implant removal four weeks later under local anesthesia [25].

In general, data suggest that large-sized OFBFs, and consequently those with greater hernia volume, are associated with a worse prognosis compared to smaller ones. This is evidenced by a significantly lower incidence of long-term complications, such as diplopia and enophthalmos, in patients with smaller OFBFs [26,27,28,29,30].

However, the clinical decision about which materials to use during repair also strongly depends on the defect size. Thus, among studies, there is an imbalance in the chosen treatments and the average defect size, representing a significant bias in evaluating the various treatment methods. A study conducted in a multivariate design could account for those inherent inequalities among the study population [26,31].

The same is true for other factors, such as patient age and a delay in surgical repair lasting longer than 2 weeks. On average, older patients have worse rehabilitation rates [5]. Older patients often experience poor oral health, malnutrition, and bad lifestyle habits, which may negatively influence the recovery and rehabilitation processes [27,32,33].

To date, no comparative studies using CT or CBT have investigated the clinical long-term outcome of Krenkel’s maxillary sinus implant compared to other commonly used materials in OFBF treatment in a multivariate design.

The aim of this study was to evaluate and compare the improvement of the orthoptic symptoms of patients after surgical repair of unilateral isolated blowout fractures by also accounting for the inherently worse prognosis of large OFF and the long-term effectiveness of Krenkel’s maxillary sinus implant in OFBF treatment relative to other materials.

## 2. Materials and Methods

The medical records of patients diagnosed with OFBF and treated at our department from January 2019 to January 2025 were retrospectively examined. Informed consent was obtained. The research was conducted in accordance with the principles outlined in the Declaration of Helsinki. For this retrospective study, approval was obtained on the 31 January 2025 from the Institutional Review Board (IRB) of the local ethics committee of Salzburg (Protocol code number: 1142/2024). 82 patients were included in this study. The preparation and writing of this study were conducted in accordance with the STROBE guidelines. The equipment was purchased from the company Wacker (Munich, BY, Germany).

### 2.1. Inclusion Criteria

Patients were included in the study if they had OFBF diagnosed with cone-beam tomography (CBT) or conventional computed tomography (CT), underwent surgical repair, and received mandatory routine check-ups.

All patients had been treated surgically using a resorbable PDS sheet or the anatomical sinus implant within two weeks of the trauma. A follow-up time of at least three months and a complete medical record were required.

### 2.2. Exclusion Criteria

Exclusion criteria for the study encompassed individuals with a history of various ophthalmologic conditions, including strabismus, amblyopia, glaucoma, age-related macular degeneration, or diabetic retinopathy. Patients with a history of orbital fractures who were surgically treated at other institutions or by alternative methods were excluded. Cases of orbital compartment syndrome, globe rupture, and concurrent midface fractures, including bilateral OFBFs or trapdoor fractures, were also omitted. Further exclusion applied to patients with OFBFs who underwent delayed repair beyond two weeks after the trauma. Finally, pediatric patients under 18 were excluded from the study.

### 2.3. Procedures

Herniated orbital tissue is released using a transconjunctival, subciliary, or subtarsal approach to the orbital floor. In cases of alloplastic repair, the OFBF is covered with a PDS sheet, while in sinus implant surgery, temporary coverage is achieved using a temporary rubber membrane. In the PDS procedure, a rubber template overlapping the defect edge is first adapted after visualization of the fracture surface. The PDS sheet is precisely adapted to this template and is then inserted into the orbit. The foil is fixed to the bony infraorbital rim or periosteum using at least two Vicryl sutures, which are placed as far apart as possible.

PDS sheets with a thickness of 0.25 mm were used. In balloon repair, access to the maxillary sinus is gained through an intraoral osteotomy of the facial maxillary sinus wall, allowing for both precise positioning of the implant and the repositioning of orbital contents that have herniated into the sinus.

An 8 mm nasal window is created in the anterior portion of the inferior nasal meatus at its lowest point to allow removal of the implant under local anesthesia. Creating a sufficiently large and smooth opening is crucial to prevent the implant from getting stuck or tearing during removal. The appropriate size of the implant is selected, which can optionally be determined by filling the maxillary sinus with sterile NaCl solution and measuring the required volume.

Before placement, the integrity of the implant is assessed by inflating it with a corresponding volume of sterile NaCl solution. Once verified, it is completely evacuated using a 20 mL syringe, ensuring proper folding. The surfaces adjacent to the medial and facial walls of the maxillary sinus are pressed together, while the portion in contact with the orbital floor bulges outward and upward.

The drain is then sealed with the provided metal stopper, and the implant is carefully introduced into the maxillary sinus, maintaining the correct orientation. This is achieved by first guiding the drain through the facial maxillary sinus window and then retrieving the tube of the implant through the nasal opening via the osteotomy window in the inferior nasal meatus. The implant is positioned within the sinus with anatomic forceps, ensuring its correct orientation and superior aspect facing superiorly.

**Figure 2:**
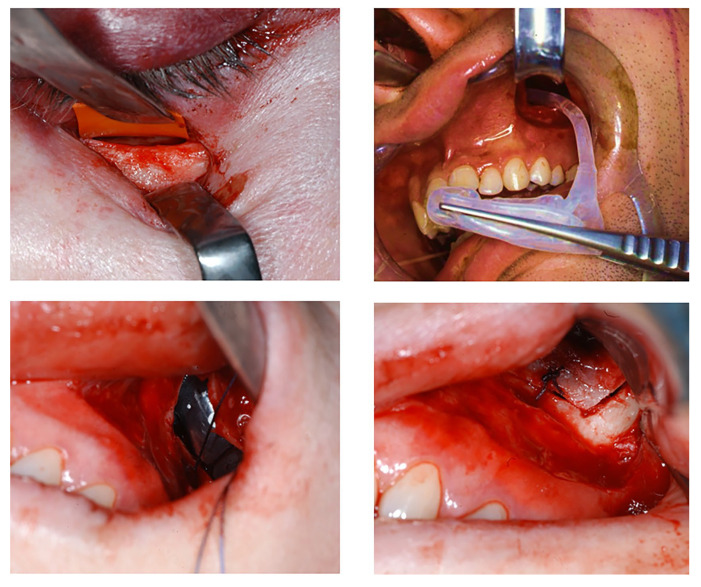
(**Top left**) placement of temporary rubber membrane to cover the orbital floor. (**Top right**) placement of the implant. (**Bottom left**) intraoral view after placement. (**Bottom right**) intraoral view after repositioning of osteotomy window.

Once in place, the stopper is removed, and the implant is filled with contrast fluid using a 20 mL syringe under direct visual control of the orbital floor. Pressure equalization is facilitated through the syringe plunger to prevent excessive pressure on the mucosa. (Figure 2) Proper filling is confirmed intraoperatively via clinical assessment and additional intraoperative CBCT imaging.

**Figure 3:**
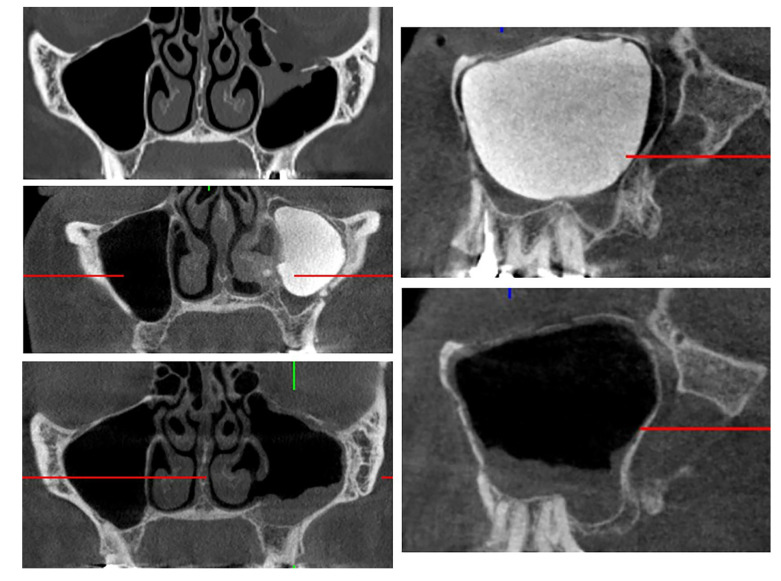
OFBF repair using the anatomical maxillary sinus implant.

The drainage tube is carefully trimmed just anterior to the nasal opening and securely sealed with the stopper. The provisional rubber membrane on the orbital floor is then removed, and the position of the orbital floor is evaluated clinically and via intraoperative CBCT (Figure 3). Finally, ocular motility is evaluated using the forced duction test in both methods. If normal mobility is confirmed, the surgical wounds are closed. Perioperative antibiotic prophylaxis is administered for 24 to 48 h. The implant is removed under local anesthesia four weeks after surgery, as described above.

### 2.4. Clinical Data

To compile all cases of OFBFs, we identified all orbital floor repair surgical procedures conducted between 2019 and 2024 based on utilizing the departments administration system Orbis (Dedalus Healthcare Group, Version 08044207.00000.DACHL, Milano, Italy).

The clinical data included age, sex, date and cause of the accident, date of operation, the size of the herniated tissue, and defect size. We also documented the materials used during surgery, the results of the orthoptic examination, including initial limitations in ocular motility, diplopia or enophthalmos, evaluation for the same parameters after surgical treatment at three to six months follow-up, and also infraorbital nerve dysfunction. We included motility impairment and diplopia as ophthalmologic symptoms and classified them based on the degree of impairment. Mild symptoms were defined as those occurring outside a 20-degree field of view, while severe symptoms were those occurring within this field.

Additionally, we noted whether revisions or other secondary procedures like balloon removal in general anesthesia were performed. We then transferred the collected data to Microsoft Excel (Microsoft, Version 2409, Redmond, WA, USA).

### 2.5. Geometric Measurements

Preoperative and postoperative ophthalmological data of patients presenting with OFBF were evaluated in relation to defect size. The OFBF area of 82 patients was measured using preoperative coronal CT or reconstructed CBCT scans. First, we exported digital imaging and communications in medicine (DICOM) data from CT and CBCT scans from our clinical imaging and documentation software DeepUnity Diagnost (Dedalus Healthcare Group, Version 1.1.1.1, Mailand, Italy). Then, we created 3D standard tessellation language (STL) models for geometric measurement by importing the data into Brainlab-Elements (Brainlab AG, Munich, Germany). Measurements were conducted using the same software to determine the fracture dimensions, including defect location and size. The Hounsfield unit was used for the segmentation threshold for CT (+1000–1600). For CT-derived STL models, the voxel size ranged from 0.2 to 0.5 mm^3^. For the CBCT STL models, the threshold for segmentation was set at the value at which the bony infraorbital rim is completely reconstructed. The voxel size for CBCT scans was set to 300 µm. The volume of herniated tissue was determined using virtual threshold segmentation based on Hounsfield units corresponding to bone and soft tissue. The segmented tissue was contiguous with the eyeball but located caudal to the orbital floor. This approach ensured that only herniated tissue was measured, excluding any contributions from sinusoidal hematoma. The measurements were verified by confirming the numbers in a second open-source software, 3D Slicer (Version 5.8.0). All measurements were done by the same person, were replicated by the second researcher, and compared for gross variances, to minimize the effect of intra-/inter-observer deviation.

### 2.6. Statistic Analysis

The data were recorded in Microsoft Excel (Microsoft, Version 2409, Redmond, WA, USA), and all statistical calculations were performed with R (R Project for Statistical Computing, Version 4.4.3) and with Statistical Package for the Social Sciences (SPSS, Version 21.0; IBM, Chicago, IL, USA).

The study cohort was characterized using means and standard deviations to describe continuous variables and proportions to describe categorical variables. The calculated OFBF areas were then analyzed, and treatment methods were compared regarding clinical outcomes, including the degree of enophthalmos, limitations in ocular motility, and infraorbital nerve dysfunction. A binary logistic regression analysis was conducted, beginning with an univariable model, followed by an adjusted multivariate model accounting for potential confounders, including age, defect size, and volume of herniated tissue. Missing values/data were excluded from the corresponding analyses, and a *p*-value < 0.05 was considered significant, whereas a *p*-value < 0.001 was considered highly significant. Model fit indices were evaluated for multivariate analyses. Model fit indices (AIC, McFadden’s R^2^, classification accuracy, and AUC) were used to evaluate the performance of the multivariate logistic regression model. Model assumptions were assessed, including multicollinearity (via variance inflation factors, VIF), and overall predictive validity. All statistical analyses were conducted and supervised by a statistician (M.G.).

## 3. Results

### 3.1. Age, Sex, and Cause of Accident

Overall, age and sex were distributed evenly across the two treatment groups. 23 (28%) of patients were female and 59 (72%) male (Table 1).

The median age was 45.2 (SD = 19.1) years. We categorized the patients using the WHO definition of geriatric patients older and adults younger than 65 but older than 18. 67 (81.7%) were adults, and 15 (18.3%) were geriatric patients, respectively. Overall, the most common cause of accidents were assaults in 28 cases (34.1%), activities of daily life in 26 cases (31.7%), comprising mostly falls and sport-related accidents in 22 cases (26.8%). However, 2 (2.4%) patients suffered a work-related accident, and 3 (3.7%) patients had a traffic accident.

Notably, the numerous bicycle accidents were categorized as sport-related, while traffic accidents only applied to trauma involving a motorized vehicle. One (1.2%) patient had committed an attempted suicide (Table 2).

**Figure 4:**
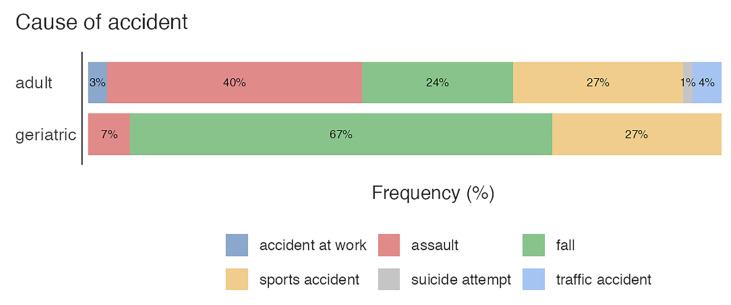
Cause of accidents among geriatric and adult patient groups.

When comparing the age groups, the adult group showed similar distribution, but the geriatric group varied, comprising predominantly of daily life activities (Figure 4).

### 3.2. Defect Size, Volume of Hernia, Initial Diplopia, Enophthalmos, and Infraorbital Nerve Impairment

**Figure 5:**
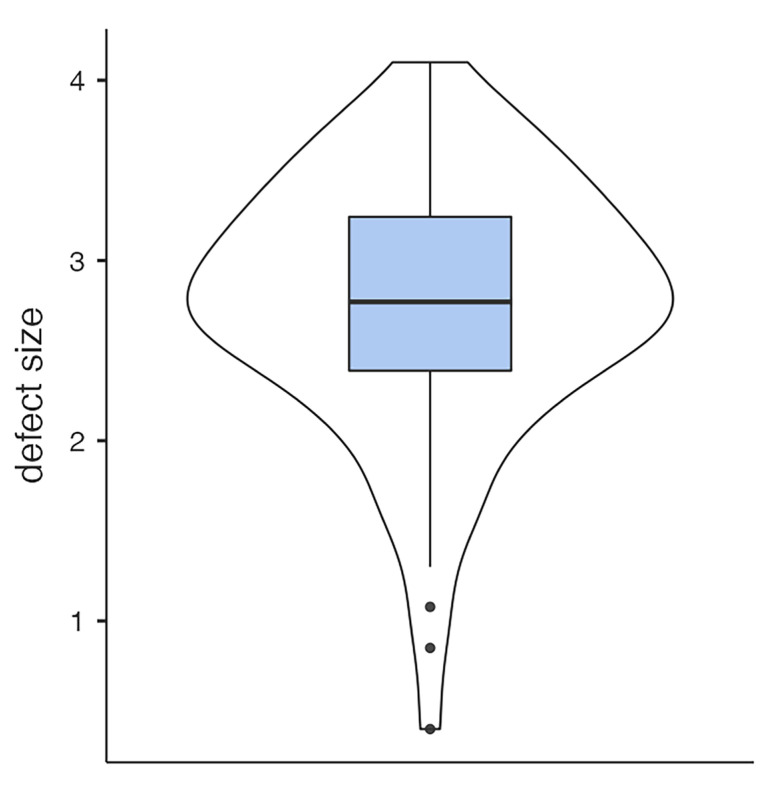
Defect size distribution.

**Figure 6:**
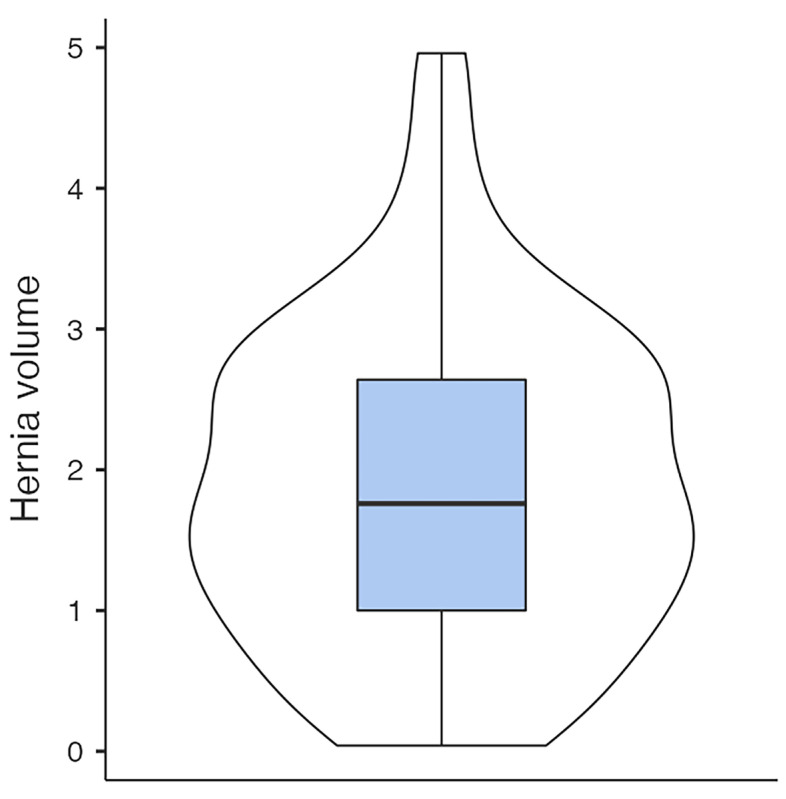
Hernia volume distribution.

The mean defect size was 2.76 (SD = 0.716) cm^2^, and the mean hernia volume was 1.9 (SD = 1.13) cm^3^ (Figure 5 and Figure 6). Initially, 20 (24.4%) patients presented with severe, and 26 (31.7%) with mild ophthalmological symptoms. Thirty-six(43.9%) showed no diplopia or impairment of motility. Nine (11%) had preoperative enophthalmos, and thirty-four (41.5%) had impaired sensibility of the infraorbital nerve. Linear regression analysis showed a strong association between the defect size of the OFBF and the volume of the herniated tissue (*p* < 0.001). Furthermore, large defect size (*p* < 0.019) and hernia volume (*p* = 0.006) were associated with preoperative diplopia. The incidence of enophthalmos showed no association with defect size or volume of the orbital hernia.

### 3.3. Treatment Methods

**Figure 7:**
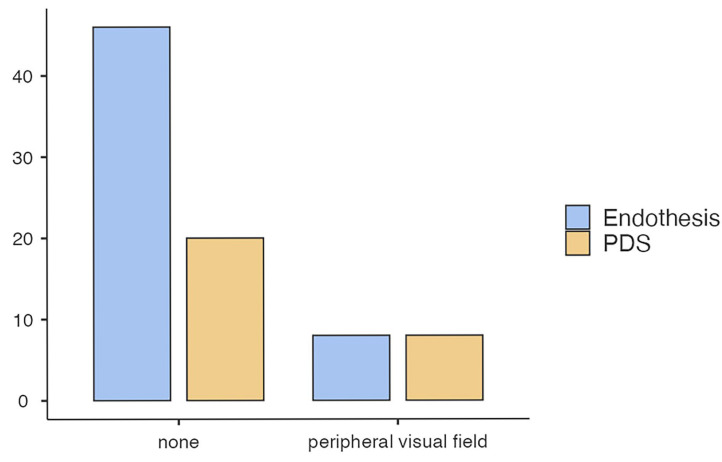
Long-term ophthalmologic symptoms among treatment groups.

At late follow-up, 8 of 54 patients (14.8%) in the implant group and 8 of 28 patients (28.6%) in the PDS group reported persistent mild ophthalmological symptoms in the peripheral visual field. No case of severe diplopia was observed in either group. The univariable analysis found no significant difference between the treatment groups. However, after adjusting for possible confounders (age, defect size, and hernia volume), PDS use was significantly (*p* < 0.01) associated with persistent ophthalmological symptoms (Table 3 and Figure 7). Model fit predictive measures and collinearity statistics (Table 3) for this binominal logistics regression model were performed.

At late follow-up three months after surgery, two patients in the antithesis group developed enophthalmos, while no cases were observed in the PDS group. However, the difference between treatment methods was not statistically significant.

The univariable analysis found no significant association between the treatment method and infraorbital nerve hypoesthesia. After adjusting for potential confounders, there was a significant correlation (*p* = 0.04) between maxillary sinus implant repair and persistent hypoesthesia of the infraorbital nerve (Table 4). Model fit along with predictive measures and collinearity statistics (Table 4) were evaluated for this binomial logistic regression model.

## 4. Discussion

OFBFs often lead to both functional and esthetic complications, including enophthalmos, diplopia, and infraorbital nerve dysfunction. The surgical intervention aims to restore orbital volume, prevent herniation of orbital contents, release incarcerated tissue, and preserve both extraocular muscle function and sensory nerve integrity. While the antral ballooning technique and PDS sheet repair are commonly used for orbital floor reconstruction, evidence suggests potential differences in their effectiveness at preventing these complications.

Enophthalmos results from an increase in orbital volume, typically caused by the herniation of periorbital tissue and transposition of the inferior rectus muscle into the maxillary sinus [6]. PDS sheets provide a rigid, resorbable scaffold that restores and maintains orbital volume while promoting the remodeling of the adjacent tissues. Studies suggest that PDS sheets are particularly beneficial in small-to-midsized orbital defects (>2–2.5 cm^2^), where moderate structural support is required to prevent late-onset enophthalmos. However, improper placement or displacement or applying the resorbable PDS sheet to too large fractures may lead to persistent volume expansion, resulting in delayed enophthalmos [8,9,10].

In contrast, maxillary sinus implant repositioning and fixation of the fractured orbital floor relies on temporary support by elevating herniated tissues via direct pressure. This method provides early-stage support but does not provide long-term structural reinforcement. We speculated that once the balloon is removed, typically after 28 days, orbital tissues may gradually descend, increasing the risk of late enophthalmos, especially in larger or comminuted fractures where natural bone healing is insufficient.

However, we did not observe an increased incidence of enophthalmos in the implant group compared to the PDS group. In accordance with our results, the evidence in the literature suggests that some ballooning techniques are well-suited for late enophthalmos prevention. Soejima et al. reported a low incidence of enophthalmos (1%) following orbital floor balloon repair [34]. Furthermore, Kashimura et al. assessed the anatomical volume of the maxillary sinus and, subsequently, the reconstruction of the orbital floor in 14 patients treated with transantral ballooning, observing stable long-term outcomes six months after surgery [35].

Being rigid yet gradually biodegradable, PDS sheets offer stable support for an extended period, thereby reducing the risk of soft tissue prolapse. However, they eventually degrade, and typically, the broken orbital floor is not routinely repositioned or supported in PDS OFBF repair, potentially leading to late enophthalmos, particularly in cases of large defects. In resorbable Mesh plates, complete degradation may take one to two years. Despite the longer degradation, the same principles apply to these biomaterials [36].

In a systematic review, Ramesh et al. observed a 5–16% incidence of enophthalmos following orbital wall repair using polydioxanone (PDS) and poly(L-lactide) implants, with PDS showing a significantly higher risk for late enophthalmos compared to the latter [37].

In a separate study, Gosau et al. observed a 3.7% incidence of enophthalmos and emphasized that in cases of OFBF repair, resorbable materials lacking osteoconductive properties, such as PDS sheets, bear a risk for late enophthalmos. They recommended that when such materials are utilized, the bony floor should be sufficiently elevated and repositioned to reduce the risk of enophthalmos [9]. However, in our clinical experience, this is not possible in many cases of larger defect sizes or comminuted OFBF.

This data suggests that, while the antral approach does not provide long-term reinforcement of the orbital floor, the repositioned bony fragments could play a role in forming a new orbital floor or, through their osteoconductive properties, aid in its regeneration [38]. These findings may reflect additional healing capacities of this technique, which could explain the excellent clinical outcomes observed in our study, particularly in terms of the low incidence of orthoptic rehabilitation and late enophthalmos even in larger fractures.

However, because the balloon applies pressure on the infraorbital nerve during repositioning, there is concern that this could impair the nerve’s regenerative capacity, especially since the infraorbital nerve is frequently involved in OFBFs. Initially, we hypothesized that the implant group might exhibit a higher incidence of persistent infraorbital nerve injuries. Our findings confirmed this hypothesis, as we observed a significantly higher incidence of mid-term hypoesthesia in the implant treatment group.

In the broader literature, infraorbital hypesthesia is reported as a relatively common complication of OFBFs, with incidence rates ranging from 12% to 53.41% [8,39,40,41,42,43,44]. It is worth noting that the anatomical maxillary sinus implant was developed using cadaveric casts, and the maxillary sinus tends to have a relatively consistent anatomical shape. In this sense, the implant can be considered analogous to a patient-specific implant (PSI). Compared to generic balloons or catheters, this anatomical conformity may help reduce focal pressure on the infraorbital nerve, potentially lowering the risk of nerve compression. However, no comparative research has evaluated the incidence of persistent infraorbital nerve hypoesthesia among antral ballooning and other OFBF repair techniques. In addition, while an average follow-up of three to four months is sufficient time for orthoptic rehabilitation, nerve healing may take significantly longer [45,46]. Peripheral nerve regeneration is often a prolonged and complex process. Following nerve injury, the distal segment of the axon and its associated myelin sheath undergo Wallerian degeneration, while the proximal axon and neuronal cell body typically remain intact. In response to the injury, Schwann cells within the distal nerve stump become activated and proliferate. These activated Schwann cells secrete a range of neurotrophic factors and cytokines that create a permissive microenvironment to support and guide axonal regrowth [47]. Recent studies on hypoesthesia and antral balloon OFBF repair are scarce. Future research regarding this technique should include further analysis of this possible complication.

Diplopia following orbital fractures often results from entrapment of the inferior rectus muscle, adhesions, or misalignment due to orbital volume changes. Surgical reconstruction should prevent muscle impingement and fibrosis, which can restrict ocular motility.

We observed persistent mild ophthalmologic symptoms in 14.8% of the implant group and 28.6% of the PDS group. Importantly, no severe symptoms were reported in either treatment cohort. These findings are consistent with those reported in the current literature, which frequently documents long-term ophthalmologic complication rates ranging from 4.9% to 45.5% [26,43,48,49,50,51].

However, it is important to note the significant variability in how ophthalmologic complications are defined across studies. Specifically, the assessment of diplopia varies, with some studies focusing on objective motility impairment while others rely on subjective reports of disruption to daily activities. Additionally, some studies utilize finger perimetric examinations, which have lower sensitivity in detecting motility disorders compared to orthoptic examinations [52]. We included orthoptically measured objective motility impairment and diplopia as ophthalmologic symptoms and classified them based on the degree of impairment. Mild symptoms were defined as those occurring outside a 20-degree field of view, while severe symptoms were those occurring within this field. This classification was based on a methodology similar to that employed by Steinmassl and Jaquiéry in their respective studies [5,26].

In our clinic, as in many others, the choice of material used during repair is largely determined by the size of the defect. This represents a significant confounding factor when evaluating the treatment method, as defect size heavily influences the outcome. Similar confounding effects may arise from other factors, such as hernia volume, patient age, and the repair timing—particularly when performed more than two weeks after the initial trauma [27,53].

We did not account for delayed surgical repair, as those cases were excluded from the study. However, we adjusted for other well-established potential confounders. After adjusting for these factors, multivariate analysis revealed significantly better outcomes concerning ophthalmological long-term rehabilitation in the implant treatment. This suggests that implant-aided repair may be a more effective method for managing OFBFs. The favorable results observed may be attributable to the nature of the treatment itself. Transantral balloon reconstruction provides a dynamic repositioning technique that does not rely on degradable materials, possibly reducing the risk of fibrosis. Thus, our data suggest that OFBF repair using the anatomic sinus implant may be a better treatment option for reducing the incidence of diplopia in larger fractures compared to PDS sheets. Both methods are effective in preventing enophthalmos. However, at least temporary infraorbital nerve disturbances are more common in patients treated with the sinus implant.

### Limitations

Given the retrospective design of this study, it was not possible to influence the selection of surgeons. The variability in surgeon skill levels and experience, particularly with differing surgical approaches, may have significantly impacted the outcomes. Additionally, variations in postoperative care and treatment protocols were not standardized, which could further contribute to the observed differences in results.

Ophthalmologic complications, such as double vision, were a critical measure of success in this study. The assessment of these complications was conducted by the Department of Ophthalmology. However, due to the retrospective design, the absence of standardized evaluation methods, as well as potential intra- and inter-observer variability, may have affected the interpretation of functional rehabilitation outcomes. This could have influenced the consistency and reliability of our findings.

While we controlled for several established clinical confounders, other unmeasured factors may still have impacted the results. Variations in surgical techniques, the skill and experience of individual surgeons, and differences in postoperative management were not systematically controlled, introducing potential sources of variability.

Another significant limitation is the unequal sample size between the two treatment groups, with 54 patients in the antithesis cohort compared to only 28 in the PDS cohort. This imbalance could affect the reliability of the results and increase the potential for statistical errors. Future studies should aim for larger, more balanced sample sizes to enhance the robustness of findings and reduce the risk of both Type I and Type II errors, ensuring more reliable and generalizable conclusions.

The relatively short postoperative follow-up period of 3–4 months is another limitation. While we cannot rule out potential changes in functional rehabilitation over time, the brief follow-up period means such changes would not significantly influence the study outcomes.

Finally, the retrospective design of the study prevents us from establishing a definitive causal relationship between the treatment modalities and the observed outcomes. Although associations between treatments and outcomes are evident, causality cannot be conclusively determined. Furthermore, since the study was conducted at a single center, the generalizability of the findings may be limited. The model’s relatively low R^2^ value suggests that unmeasured variables may contribute to the observed effects. Future studies with larger, more diverse samples and additional clinical or psychosocial predictors could improve model accuracy and explanatory power. To confirm these results and draw more definitive conclusions, prospective randomized controlled trials and multicenter studies are needed. We plan to expand the dataset in future research. This would allow for the application of artificial intelligence to assess positive or negative prognostic factors [54,55].

## 5. Conclusions

The anatomical maxillary sinus implant has been used in our department for more than 40 consecutive years. Although its use requires a complex procedure with numerous intricate steps, an additional approach to the maxillary sinus and the need for removal four weeks later, the individual support of the fractured orbital floor facilitates healing using the osteoconductive properties of the bone. In addition, there is no foreign body reaction or long-term scar inducing degradation after removal. Our findings suggest that the anatomical maxillary sinus implant is a valid and effective tool for orbital floor repair and ocular rehabilitation following OFBFs.

## Figures and Tables

**Table 1:** Sex distribution.

Sex	Counts	% of Total	Cumulative%
female	23	28.0 %	28.0%
male	59	72.0 %	100.0%

**Table 2:** Causes of accident.

Cause of Accident	Counts	% of Total	Cumulative%
accident at work	2	2.4%	2.4%
assault	28	34.1%	36.6%
fall	26	31.7%	68.3%
sports accident	22	26.8%	95.1%
suicide attempt	1	1.2%	96.3%
traffic accident	3	3.7%	100.0%

**Table 3:** Binominal logistic regression of persistent ophthalmologic symptoms comparing the treatment groups (*n* = 82).

Predictor	Estimate	95% Confidence Interval		SE	Z	*p*	Odds Ratio
		Lower	Upper				
Intercept	−8.24739	−12.9971	−3.4977	2.4234	−3.403	<0.001	2.62 × 10^−4^
Implant or PDS:							
PDS–Implant	2.0076	0.5134	3.5019	0.7624	2.633	0.008	7.45
Age	0.00658	−0.027	0.0402	0.0171	0.384	0.701	1.01
Defect size	1.3175	−0.092	2.727	0.7191	1.832	0.067	3.73
Hernia volume	0.82226	0.1121	1.5324	0.3623	2.269	0.023	2.28

Model fit: AIC = 68.4, McFadden’s R^2^ = 0.275, accuracy = 0.84, AUC = 0.839. Classification threshold set at 0.5, VIF: [1.02–1.34]. All variance inflation factors (VIF) were below 1.35, indicating no significant multicollinearity.

**Table 4:** Binominal logistic regression of persistent infraorbital nerve dysfunction comparing the treatment groups (*n*= 82).

Predictor	Estimate	95% Confidence Interval		SE	Z	*p*	Odds Ratio
		Lower	Upper				
Intercept	−3.13834	−6.1402	−0.1364	1.5316	−2.049	0.04	0.0434
Implant or PDS:							
PDS–Implant	−0.2914	−1.5195	0.9367	0.6266	−0.465	0.642	0.7472
Age	0.00484	−0.0236	0.0333	0.0145	0.333	0.739	1.0049
Defect size	0.21674	−0.9002	1.3337	0.5699	0.38	0.704	1.242
Hernia volume	0.6342	−5.04 × 10^−4^	1.2689	0.3238	1.958	0.05	1.8855

Model fit: AIC = 92.3, McFadden’s R^2^ = 0.112, accuracy = 0.802, AUC = 0.705. Classification threshold set at 0.5, VIF: [1.02–1.57]. All variance inflation factors (VIF) were below 1.58, indicating no significant multicollinearity.

## Data Availability

The data presented in this study are available on request from the corresponding author. For privacy and ethical restrictions.
